# Genotyping transposable element insertions and their novel application in gene mapping strategies for Alzheimer’s disease (AD) endophenotypes

**DOI:** 10.1002/alz.085920

**Published:** 2025-01-03

**Authors:** Muralidharan Sargurupremraj, Qiong Yang, Xueqiu Jian, Jayandra J. Himali, Sudha Seshadri

**Affiliations:** ^1^ University of Texas Health Science Center at San Antonio, San Antonio, TX USA; ^2^ Boston University, Boston, MA USA; ^3^ Boston University School of Public Health, Boston, MA USA; ^4^ The University of Texas Health Science Center at San Antonio, San Antonio, TX USA; ^5^ Department of Population Health Sciences, University of Texas Health Sciences Center, San Antonio, TX USA; ^6^ Glenn Biggs Institute for Alzheimer’s & Neurodegenerative Diseases, University of Texas Health Science Center, San Antonio, TX USA; ^7^ Department of Neurology, University of Texas Health Sciences Center, San Antonio, TX USA; ^8^ Boston University and the NHLBI’s Framingham Heart Study, Boston, MA USA

## Abstract

**Background:**

Consortium‐wide studies of volumetric brain imaging measures with single‐nucleotide polymorphisms (SNPs) have revealed numerous disease‐risk SNPs and emphasized the significance of brain imaging phenotypes as preclinical markers (endophenotypes) for Alzheimer’s disease (AD). Nevertheless, the bulk of these risk variants are in genomic regions that govern multiple genes, posing major challenges in fine‐mapping strategies. Evolutionarily conserved transposable elements are master regulators of gene expression, and by studying these endogenous gene regulatory units in relation to AD endophenotypes, we aimed to better identify the disease‐causal gene.

**Method:**

Within the Trans‐Omics for Precision Medicine Program (TOPMed) initiative, whole‐genome sequencing data (WGS) of the Framingham Heart Study individuals were used for genotyping polymorphic transposable element insertions (pTEIs). Fitting a whole‐genome regression model, we tested the association of the genotyped pTEIs with brain‐imaging measures (N = 2,472) (white matter hyperintensity, WMH; hippocampal volume, HV; total‐brain volume, TBV) and general cognitive function, GCF (N = 2,765). We performed i) single‐variant analyses (minor allele frequency ≥ 1%) and ii) region‐based burden tests per gene collapsing rare pTEIs overlapping with canonical transcripts and regulatory marks. We examined the colocalization evidence between the pTEIs and SNPs identified in genome‐wide association studies (GWAS) for our traits of interest, thus enhancing the fine‐mapping of disease‐risk SNPs.

**Result:**

By studying the polymorphic nature of TE insertions in relation to brain‐imaging and cognitive traits, we identify evidence for multiple putative disease‐causal genes, albeit suggestive. Notably, this includes genes (*BACH2, SPATA5*) implicated in autoimmune and neurodevelopmental disorders, now showing pTEI‐based predisposition to cognitive decline and hippocampal atrophy. WMH and TBV risk SNPs exhibit colocalization evidence with pTEIs involved in the feature elongation of *ECHDC3* and *CRHR1*, respectively, potentially leading to loss of gene function. Interestingly, the inclusion of ECHDC3 gene loss‐of‐function variants in burden test analysis (UK Biobank) shows association with brain maturation and ageing markers.

**Conclusion:**

Our pragmatic approach using existing WGS data to genotype endogenous regulatory units for studying genetic underpinnings of preclinical AD markers identifies putatively novel disease‐causal genes with clear biological function. As a proof of concept, our approach highlights the scope of extending to other cohorts and AD related neurodegenerative disorders.

